# Positive, Open, Proud: an adapted disclosure-based intervention to reduce HIV stigma

**DOI:** 10.3389/fgwh.2024.1469465

**Published:** 2024-11-27

**Authors:** Tiffany Chenneville, Kristin Kosyluk, Kemesha Gabbidon, Molly Franke, Dylan Serpas, Jerome T. Galea

**Affiliations:** ^1^ Department of Psychology, University of South Florida, St. Petersburg, FL, United States; ^2^ Department of Pediatrics, University of South Florida, Tampa, FL, United States; ^3^ Perinatal HIV Research Unit, School of Clinical Medicine, University of Witwatersrand, Johannesburg, South Africa; ^4^ Department of Mental Health Law and Policy, University of South Florida, Tampa, FL, United States; ^5^ Department of Global Health and Social Medicine, Harvard Medical School, Boston, MA, United States; ^6^ Department of Epidemiology, Harvard T.H. Chan School of Public Health, Boston, MA, United States; ^7^ Department of Psychology, University of South Florida, Tampa, FL, United States; ^8^ Department of Social Work, University of South Florida, Tampa, FL, United States

**Keywords:** HIV, disclosure, women, social stigma, psychosocial intervention, mental disorders

## Abstract

HIV stigma among people living with HIV (PLWH) is well documented and linked to adverse physical and mental health outcomes among this population. Further, stigma may affect HIV disclosure decisions, which has important individual and public health implications. For women, HIV stigma and disclosure may be compounded by gender-based discrimination and violence. Despite the ill effects of HIV stigma, particularly for women, few evidence-based disclosure interventions to reduce stigma among PLWH exist. However, there is strong evidence for the efficacy of Honest, Open, Proud (HOP), a disclosure-based stigma-reduction intervention for people with mental illness. Given that mental illness and HIV are similar in that they are both stigmatized yet concealable conditions, we propose using the ADAPT-ITT model to adapt HOP into Positive, Open, Proud, a disclosure-based stigma-reduction intervention for PLWH, describing its unique potential for women living with HIV.

## 1 Introduction

In his seminal work, Irving Goffman (1) defined stigma as a social phenomenon that discredits and devalues individual characteristics. Since then, defining and conceptualizing stigma has presented numerous challenges given the broad range of circumstances to which stigma applies (2), leading researchers to approach stigma from different theoretical lenses based on their respective fields. Nonetheless, there is considerable overlap in the types of stigmas that have been studied. Here, we focus on public stigma and self-stigma.

Public stigma, also referred to as social stigma, personal stigma, public prejudice, labeling, marginalization, or cultural bias, encompasses the stereotypes (cognitions or beliefs) and corresponding prejudice (emotional reactions) of the general public toward individuals who are marginalized, and the resulting discrimination of stigmatized group members (3).

Self-stigma, also known as internalized stigma, internalized shame, self-devaluation, or internalized prejudice involves individuals who occupy marginalized identities accepting and integrating the negative attitudes, beliefs, and stereotypes of the broader society (4). Stigma can be intersectional in nature (5), meaning several forms of interlocking forms of stigma (e.g., gender-based stigma, race-based stigma, HIV stigma) manifest simultaneously to produce a heightened and nuanced experience. Intersecting stigmas often reinforce power dynamics, leading to varied impacts based on societal hierarchies and differential stigma experiences. For example, a Black woman living with HIV [LWH] may experience stigma differently than a white man LWH. Both public and self-stigma contribute to label avoidance and concealment, further limiting access to social support systems that may help alleviate stressors. Further, public stigma poses a serious public health concern given its relationship with various negative outcomes, including residential segregation, educational and health disparities, social isolation, and diminished quality of life (6), thus, perpetuating inequality and marginalization. Public stigma is directly linked to self-stigma when societal beliefs are internalized (7), leading to maladaptive psychological responses, such as rumination and depression, as well as behavioral outcomes, like substance use (6). Two of the most highly stigmatized conditions are HIV and mental illness (8). In this paper, we explore the similarities and differences between mental illness and HIV as stigmatized conditions; provide an overview of existing HIV stigma interventions and their limitations; describe an evidence-based disclosure intervention to reduce mental illness stigma, Honest, Open, Proud (HOP); describe how HOP might be adapted to provide a novel disclosure-based HIV stigma-reduction intervention, Positive, Open, Proud (POP); and explain why POP may be particularly useful for women living with HIV (WLWH) given the impact of gender-based power dynamics on disclosure decisions.

## 2 Similarities and differences between mental illness and HIV as stigmatized conditions

Stigma is broadly understood to be undesirable and harmful, regardless of the health condition, and is often described in monolithic terms, which may belie important differences between stigmatized conditions. While mental illness and HIV stigma share some similarities, there are crucial differences in HIV that necessitate unique, targeted interventions (8). Here, we consider the key similarities and differences between mental illness and HIV that affect the manifestation of stigma.

### 2.1 Similarities

The first similarity between HIV and mental illness is their potential for concealment (9). Although there are exceptions (e.g., untreated HIV resulting in Kaposi sarcoma, a rare cancer that is a hallmark of advanced HIV disease, and certain severe mental illnesses involving disorganized speech or involuntary motor movements), most PLWH and/or a mental illness can hide their condition from others, thus avoiding the resulting stigma (9). Of note, although people with less visible chronic health conditions may report less stigma, the magnitude of its adverse effects on mental health may be greater than those with visible conditions (10). A second similarity is that both HIV and mental illness tend to be chronic and require regular intervention (whether medication, self-care strategies, or other therapeutic approaches) to maintain wellness. Left untreated, both can cause serious morbidity and mortality. A third commonality between HIV and mental illness is that both may invoke negative perceptions of character, for example, promiscuity or immorality for PLWH and weakness and chosen fragility for persons with mental illness.

### 2.2 Differences

As an infectious disease, the first significant difference between HIV and mental illness is that HIV, unlike mental illness, is both acquired and can be transmitted to others. While emerging evidence suggests genetic correlates for some serious mental disorders (e.g., schizophrenia), this differs from a virus that can be isolated and passed to others. Further, how HIV is transmitted, primarily through sexual contact (though not always, as in the case of acquiring HIV via a blood transfusion or during birth), is also frequently stigmatized especially same-sex sexual activity, forced/non-consensual sex, commercial sex work, or sex outside an established primary relationship. These differences in stigma are especially important when disclosing HIV to others because the process may simultaneously reveal the HIV status of someone other than the discloser and/or personal information (11). For example, disclosing living with HIV invariably conjures the question “How did you get it?” and may lead to the disclosure of other people (e.g., sex partner, mother) or reveal past trauma (e.g., rape, sexual assault) or other information that might generally be kept private. Finally, HIV disclosure also frequently involves the revelation of other stigmatized identities (e.g., sexual orientation, gender identity) or behaviors (e.g., same-sex sexual partnerships, substance use including intravenous drug use) (12) for which there may be magnified disclosure consequences.

## 3 HIV stigma and disclosure among women: unique considerations

WLWH often face significant compounded stigmas based on their multiple marginalized identities which are characterized by power dynamics and gender-based discrimination resulting in poorer mental health and quality of life (13, 14). Societal norms and cultural expectations about women's behavior, including sexual conduct and fidelity, may result in unfair blame and increased moral and social judgement, exacerbating the HIV stigma they face (14, 15). Women may have less access to healthcare resources due to gender biases in healthcare systems, restricted mobility, childcare obligations, and other gender-related barriers (16). HIV stigma can further limit women's ability to seek and receive appropriate medical care and impact their ability to fulfill caretaking roles, affecting both their well-being and that of those they care for, which is particularly problematic given that women often bear the primary responsibility for caregiving within families. Fear of HIV stigma can strain social networks, reducing the support women receive from friends, relatives, and community members.

The interplay between HIV stigma and HIV disclosure decisions can be complicated by gender-based power dynamics. Women may face increased risks of interpersonal violence or abandonment if they disclose their HIV status (17). The fear of physical, emotional, or economic abuse from partners or family members can deter women from disclosing their HIV status. Many women, especially in patriarchal societies, may be financially dependent on their partners or families. Disclosure of HIV status could jeopardize their financial security and that of their children, making them more vulnerable to poverty and homelessness. In a recent study of 118 pregnant WLWH in Ghana, fear of loss of financial support was the most common reason for nondisclosure of HIV status (18). Women of childbearing age face specific concerns related to pregnancy and childbirth. Disclosure of HIV status is crucial for accessing prevention of mother-to-child transmission services, but fear of blame and stigma can deter women from seeking these and other healthcare services (19, 20).

## 4 Evidence-based disclosure interventions to reduce self-stigma

In the mental health stigma reduction field, two predominant approaches are used to reduce self-stigma: (1) education-based and cognitive restructuring strategies teaching people ways to counter stereotypes by providing facts that counter myths (21), and (2) disclosure-based strategies, which recognize that due to the concealable nature of the stigmatized identity of mental illness, people with mental illness face the choice of whether to disclose or conceal (1). Disclosure-based self-stigma reduction strategies rest on research showing that people with concealable stigmatized identities (e.g., mental illness, HIV) who identify with peers and are publicly out with their stigmatized identity may experience better mental and physical health as well as better relationships (21–23).

Separate bodies of literature document HIV stigma reduction interventions (24, 25) and HIV disclosure uptake interventions (26, 27) for PLWH. Existing HIV stigma reduction interventions have utilized primarily psychoeducation-based approaches (28), shown wide variability in producing change in targeted outcomes (24), and targeted a single socio-ecological level (e.g., individual level) focusing on one domain of stigma (29). Structural HIV stigma reduction interventions such as economic strengthening, social empowerment, and antiretroviral treatment provision coupled with individual components (e.g., health education) have been most effective in reducing self-stigma in PLWH (30). Few individual and relational interventions are documented, and existing studies have not found significant reductions in self-stigma (30). Further, few HIV stigma reduction interventions that possess strong methodological rigor have considered intersectional stigmas in HIV (25). Meanwhile, available HIV disclosure uptake interventions are narrow in scope, often focusing on a specific form of disclosure (e.g., sexual partner disclosure only). These interventions are characterized by variable content and operationalization of disclosure (26). There remains a need to not only advance the quality of methodological rigor in HIV stigma reduction and disclosure uptake interventions but also to combine these complementary approaches, which is not common practice (30).

The few combined HIV stigma and disclosure-targeted interventions available have demonstrated mixed efficacy, with one randomized control entertainment-education intervention finding positive changes in disclosure attitudes, self-efficacy, and intentions to disclose, but no changes in internalized stigma in Black women in the Southern United States (31). Similarly, a randomized control trial of a community-based culturally informed motivational interviewing style intervention for Black PLWH found no changes in internalized stigma and disclosure across a 13-month period (32). Considering the complexities surrounding HIV stigma reduction and disclosure uptake interventions, it is essential to explore innovative approaches that hold promise in addressing these challenges and fostering positive outcomes for PLWH.

## 5 Honest, open, proud: a model evidence-based disclosure intervention to reduce self-stigma

A model evidence-based disclosure intervention to reduce mental illness self-stigma, Honest, Open, Proud (HOP) was developed using community-based participatory research methods to directly target key mediators of mental illness self-stigma and its harmful impact (33). The core intervention consists of three lessons by two trained peer facilitators (individuals also living with the stigmatized condition) in groups of six to ten participants. Lesson delivery can occur in a one-day session, in two half-day sessions, or over the course of three consecutive weeks for two hours per week. As described below, each of the three lessons is focused on developing a disclosure-related skill, followed by a booster session.

### 5.1 Lesson 1: evaluate the pros and cons of disclosing

Motivators have important and dynamic effects on disclosure (34). HOP teaches participants to identify the costs and benefits of disclosure, which are likely to vary by setting or recipient (e.g., disclosing to family vs. friends, schoolmates, partners). Hence, program participants learn the skills to think through potential risks and complete cost-benefit analyses in terms of specific settings or people. Participants learn that disclosure is not a “one size fits all” endeavor but must be tailored to each individual.

### 5.2 Lesson 2: learn ways to identify safe people to disclose to

Given the importance of social support in disclosure, HOP participants learn strategies to test the likelihood that specific people in specific settings (e.g., a medical provider in a clinic, or a classmate at school) are likely to react positively to disclosure.

### 5.3 Lesson 3: craft a personal disclosure story

Rooted in research demonstrating that the quality of the story and personal narrative is associated with decreased self-stigma (35), participants learn the elements of an effective disclosure story that they can use to write their own story for future disclosure.

### 5.4 Booster

A fourth booster session is delivered a month post-intervention for participant reflection on any disclosure experiences since completing lessons 1 through 3, and to provide ongoing peer support.

### 5.5 HOP research

An accumulating evidence base, including multiple randomized controlled trials, suggests HOP is effective in reducing self-stigma and related outcomes among people living with mental illness. Table 1 summarizes the details of existing randomized controlled trials of HOP for mental illness. A recent meta-analysis of the literature on HOP revealed statistically significant positive effects on stigma stress as well as modest differences in self-stigma and depression (37). HOP has been adapted for other stigmatized conditions (e.g., suicide survivorship and Tourette's Syndrome) and special populations (e.g., Veterans) (https://hopprogramorg). For example, a pilot study of HOP for suicide survivors found significant effects among HOP participants relative to a control group for two measures of self-stigma, depression, and self-esteem (42).

**Table 1:** Existing efficacy studies of HOP for people with mental illness.

First author	Study design	Population	Mental illness type	Methods	Efficacy outcomes
Corrigan et al. (36)	Randomized controlled trial; 3-session COP program (*n* = 51) and waitlist control (*n* = 51) to reduce self-stigma	126 adults who self- identified with mental illness and reported shame with that identity	Participants responding positively to the following two questions: (1) “Do you see yourself as a person with mental illness or mental health challenges?” and (2) “Do you feel some sense of shame because of the mental illness or mental health challenges?”	Self-reported self-stigma, stigma stress, and depression	Compared to control group (no change), the intervention group participants experienced the following outcomes at pos*t*-test and follow up: significantly diminished self- stigma, significant reductions in stress related to stigma, and significantly more resources to cope with stigma.
Rüsch et al. (37)	Pilot randomized controlled trial with 100 adults assigned to either COP (*n* = 50) or TAU (*n* = 50)	100 adults who self-reported current Axis 1 or Axis II disorders according to DSM-IV criteria and moderate disclosure-related distress	Depressive disorder: COP (56%), TAU (64%); Bipolar disorder: COP (18%), TAU (22%); Schizophrenia spectrum disorder: COP: (32%), TAU (22%)	Self-reported self-stigma, empowerment, secrecy, perceived benefits of disclosure, cognitive appraisal of stigma, disclosure-related self-efficacy, and depression	Compared to TAU group (no change), the intervention group participants experienced the following outcomes at pos*t*-test and follow up: significant reductions in stress related to stigma, less distress at idea of disclosure, more perceived benefits of disclosure, and less perceived need to keep their identity a secret.
Conley et al. (38)	Randomized controlled trial with 118 college students assigned to either HOP-C (*n* = 63) or waitlist control (*n* = 54)	118 undergraduate students across three campuses	College students who self-identified as having a mental illness or mental health challenge. Clinically elevated depression: total sample: 58.5%; clinically elevated anxiety: 69.2%	Self-reported self-stigma, stigma appraisals (stress and coping), self-efficacy about disclosure or secrecy	Compared to the control group (no change), the intervention group participants experienced the following outcomes at pos*t*-test and follow up: reduced self-stigma (particularly harm from self-applied stereotypes), increased appraisals of perceived resources to cope with stigma-related stress, and increased self- efficacy about disclosure.
Mulfinger et al. (39)	Randomized controlled trial with 98 participants assigned to either HOP (*n* = 49) or TAU (*n* = 49)	98 predominantly inpatient adolescent participants who self- reported one or more psychological disorders and at least a moderate level of disclosure- related distress	Depressive disorder: HOP (64%), TAU (58%), Anxiety disorder: HOP (19%), TAU (17%)	Self-reported stigma stress, health-related quality of life, self-esteem, optimism, disclosure-related distress, hopelessness, self-stigma, intentions to seek help for mental health problems, recovery, secrecy, social withdrawal, and depressive symptoms.	Compared to treatment as usual group (no change), the intervention group participants experienced the following outcomes at pos*t*-test and follow up: improvements in self-stigma, appraisals of stigma as stressful, empowerment, disclosure-related distress, secrecy, social withdrawal, help-seeking intentions, attitudes to disclosure, stage of recovery, quality of life, and depressive symptoms at post- intervention and three-week follow-up.
Qin et al. (40)	Randomized controlled trial with 135 participants assigned to either HOP (*n* = 68) or waitlist control (67)	135 Chinese people living with serious mental illness	Schizophrenia: HOP (60.3%), control (56.7%); Bipolar: HOP (11.8%), control (7.5%); Depression: HOP (25.0%), control (25.4%); Adjustment disorder: HOP (0%), control (1.5%); Anxiety disorder: HOP (2.9%), control (6.0%); Mood disorder: HOP (0%), control (1.5%)	Self-reported self-efficacy disclosure, and self-stigma	Compared to a waitlist control group, the intervention group experienced decreased self-stigma, specifically the application of stereotypes to the self.
Modelli et al. (41)	Randomized controlled trial with 61 patients with mood disorders assigned to either HOP (*n* = 30) or psychoeducational control (*n* = 31)	61 patients (31 diagnosed with depression and 30 diagnosed with bipolar disorder) based on DSM-V criteria.	Intervention group: 50% bipolar; Control: 48.4% bipolar	Self-reported quality of life, self-esteem, perceived benefits of coming out, authenticity, self-stigma, internalized stigma, stigma stress, and barriers to access care,	Compared with an unstructured psychoeducation control group, the intervention groups (depression and bipolar) did not present a significant change regarding the decision to disclose their diagnosis. Individuals in the depression group showed a decrease in the perception of stigma as a stressor. Improvements were seen for both groups (depression and bipolar) on sense of authenticity.

COP, coming out proud; TAU, treatment as usual; HOP-C, honest, open, and proud-college; DSM, diagnsotic and staitstical manual.

## 6 Adapting HOP for HIV: Positive, Open, Proud

Given the similarities between mental illness and HIV, as described above, and existing research in support of the effectiveness of HOP for facilitating disclosure decisions and reducing stigma stress among people with mental illness (37), an adaptation of HOP for HIV offers a promising and innovative disclosure-based self-stigma reduction intervention. Like mental illness, HIV is a concealable, stigmatized identity. Therefore, PLWH face the choice of whether, how, and when to discuss their disease status with others, considering the potential benefits and costs of HIV disclosure. See Table 2. Theoretically, the decision-making and action of mental health disclosure is like HIV disclosure as it involves antecedent goals, outcome expectancies, intention, and action planning. Therefore, we are adapting the HOP curriculum for PLWH - Positive, Open, Proud (POP) - using the ADAPT-ITT model (43), which employs a systematic process for adapting evidence-based interventions using 8 steps: (1) assessment to obtain a comprehensive understanding of the target population (in this case, PLWH); (2) decision, which involves selecting an intervention (in this case, HOP) and deciding whether to adopt or adapt (in this case, adapt); (3) adaptation by using a pretested methodology to understand better how to adapt HOP for PLWH; (4) production, which requires creating an adaptation plan and determining goals; (5) topical experts, which involves obtaining substantive content and technical assistance; (6) integration, which involves integrating all forms of information; (7) training all personnel; and (8) testing via a pilot study to assess adaptation efficacy.

**Table 2:** Potential benefits and costs of disclosing HIV status.

Potential benefits	Potential costs
Eliminate worry about hiding HIV status from friends, family, co-workers, & others	Negative response/disapproval in response to disclosure
Free to engage in day-to-day affairs	Stigmatization/discrimination
Can be honest with supervisors/bosses/co-workers when requesting time off or missing work to attend medical appointments	Gossip
Social support	Exclusion from social events
May meet others who are also HIV positive	Concern about others’ perceptions
Eliminate concern about HIV criminalization laws	Negative impact on dating/intimate relationships

Four of the authors of this article have HIV expertise and work closely with affected communities and community advisory groups comprised of PLWH, thus facilitating Step 1 (Assessment). By collaborating with another author who has conducted extensive research on mental illness stigma-reduction through self-disclosure, we decided to adapt HOP for HIV (Step 2). In preparation for Steps 3–8, a person with lived experience with HIV assisted with an initial desk adaptation of the HOP curriculum to POP. Like HOP, POP consists of three lessons to help PLWH (1) evaluate the pros and cons of disclosure, (2) select ways to disclose and to whom, and (3) develop ways to tell their story. A fourth booster session one-month post-intervention is designed to provide ongoing peer support and allow participant reflection on any disclosure experiences following Lessons 1–3.

We plan to partner with an HIV community advisory board (CAB) comprised of a diverse group of PLWH to ensure, consistent with ADAPT-ITT guidelines, the target population/key stakeholders are involved in all phases of the adaptation process. Specifically, we will use a pretested methodology to understand better how to further adapt HOP to POP (Step 3), create an adaptation plan and determine goals (Step 4), engage topical experts to provide substantive content and technical assistance (Step 5), and to integrate all forms of information (Step 6). We will then train two peer facilitators with lived experience with HIV (Step 7). Finally, we will pilot POP with PLWH to determine its efficacy as a disclosure-based self-stigma reduction intervention (Step 8).

## 7 Potential benefits of POP for women living with HIV

Given the impact of gender dynamics on perceptions of HIV and decision-making around sexual health (44, 45) a need exists for HIV disclosure interventions for WLWH (18), especially interventions that prioritize choice (46). Therefore, we believe POP may be particularly useful for women as an HIV disclosure-based stigma-reduction intervention.

Gender-based power imbalances may affect women's ability to disclose their HIV status safely and confidently (17). POP may help provide women with the tools and support needed to navigate these power dynamics and make informed decisions about disclosure. Disclosure of HIV status can lead to better health outcomes by facilitating access to healthcare, support services, and social networks. For women, this is particularly important as they may have additional health needs related to reproductive health, pregnancy, and childcare. Reducing stigma can encourage women to seek and adhere to treatment, ultimately improving their health and quality of life. By providing knowledge, skills, and support to make autonomous decisions about their health and lives, POP may empower women, which may help them negotiate safer sexual practices, improve their self-esteem, and reduce their dependency on partners who may be unsupportive or abusive.

The association between HIV and gender-based violence is well documented (47). POP recognizes that HIV disclosure can sometimes trigger violence if not handled sensitively and, therefore, does not encourage disclosure but, rather, helps PLWH explore disclosure decisions. For women, this includes the exploration of safe and supportive options for disclosure.

## 8 Conclusion and future directions

Despite some unique differences, there are many similarities between HIV stigma and mental illness stigma. Given the strong evidence for the efficacy of HOP as a disclosure-based stigma-reduction intervention for people living with mental illness and other conditions, adapting this intervention to reduce stigma among PLWH shows promise, especially given the lack of evidence-based disclosure interventions to reduce HIV self-stigma. Therefore, we are using the ADAPT-ITT Model (43) to develop and pilot Positive, Open, Proud as a disclosure-based stigma-reduction intervention for PLWH. We believe POP may be particularly useful for addressing the unique challenges faced by WLWH, specifically related to the impact of gender-based discrimination, violence, and power dynamics on HIV stigma and disclosure.
